# Clinical Importance of the Heel Drop Test and a New Clinical Score for Adult Appendicitis

**DOI:** 10.1371/journal.pone.0164574

**Published:** 2016-10-10

**Authors:** Shin Ahn, Hyeji Lee, Wookjin Choi, Ryeok Ahn, Jung-Suk Hong, Chang Hwan Sohn, Dong Woo Seo, Yoon-Seon Lee, Kyung Soo Lim, Won Young Kim

**Affiliations:** 1 Department of Emergency Medicine, University of Ulsan College of Medicine, Asan Medical Center, Seoul, Korea; 2 Department of Emergency Medicine, University of Ulsan College of Medicine, Ulsan University Hospital, Ulsan, Korea; University Hospital Oldenburg, GERMANY

## Abstract

**Objective:**

We tried to evaluate the accuracy of the heel drop test in patients with suspected appendicitis and tried to develop a new clinical score, which incorporates the heel drop test and other parameters, for the diagnosis of this condition.

**Methods:**

We performed a prospective observational study on adult patients with suspected appendicitis at two academic urban emergency departments between January and August 2015. The predictive characteristics of each parameter, along with heel drop test results were calculated. A composite score was generated by logistic regression analysis. The performance of the generated score was compared to that of the Alvarado score.

**Results:**

Of the 292 enrolled patients, 165 (56.5%) had acute appendicitis. The heel drop test had a higher predictive value than rebound tenderness. Variables and their points included in the new (MESH) score were pain migration (2), elevated white blood cell (WBC) >10,000/μL (3), shift to left (2), and positive heel drop test (3). The MESH score had a higher AUC than the Alvarado score (0.805 vs. 0.701). Scores of 5 and 11 were chosen as cut-off values; a MESH score ≥5 compared to an Alvarado score ≥5, and a MESH score ≥8 compared to an Alvarado score ≥7 showed better performance in diagnosing appendicitis.

**Conclusion:**

MESH (migration, elevated WBC, shift to left, and heel drop test) is a simple clinical scoring system for assessing patients with suspected appendicitis and is more accurate than the Alvarado score. Further validation studies are needed.

## Introduction

Acute appendicitis is one of the most common abdominal surgical emergencies presenting at the emergency department (ED) [1–3]. Despite the increasing availability of ultrasonography and computed tomography (CT), clinical examination remains the cornerstone of the diagnostic process when patients present with right lower quadrant pain. Recent guidelines recommend the establishment of local pathways for the diagnosis of acute appendicitis and note that the combination of clinical and laboratory findings of pain characteristics, tenderness, and laboratory evidence of inflammation identify most patients with suspected appendicitis [4]. Physical examination may reveal signs of peritoneal irritation in the right lower quadrant or diffusely. In addition, other symptoms such as obturator sign, psoas sign, or Rovsing’s sign may be associated with appendicitis depending on the location of the inflamed appendix. However, these indications are only weakly predictive of appendicitis [5]. The heel drop test has been shown to be superior to the old rebound test for detecting intraperitoneal inflammation since it is more objective and less subject to misinterpretation [6]. However, only one study in Turkey has been performed on the usefulness of the heel drop test as a clinical indication of acute appendicitis [7]. Other diagnostic strategies include the use of scoring systems, of which the Alvarado score, derived from retrospectively collected data from 305 adult patients in the mid-1980s, is the best known clinical prediction rule for estimating the risk of appendicitis [8–12]. This score is calculated from symptoms, physical examination, and basic laboratory data and assigns a score from 0 to 10. The original study of this system reported a sensitivity of 81% and specificity of 74% in identifying patients who needed an appendectomy, and subsequent validation studies have showed variable performances of this score [13–15]. The modified Alvarado score uses the same value categories without the shift to left of leukocytosis, ranging from a score of 0 to 9 [16]. Patients with an Alvarado score <5 or a modified Alvarado score <4 are considered to be at low risk for appendicitis.

The primary aim of our present study was to evaluate the accuracy of the heel drop test as a clinical factor in acute appendicitis. We compared its performance with that of other well-known physical examination findings of appendicitis. We also tried to develop a new clinical score for adult appendicitis based on the heel drop test as a variable, and tried to compare the reliability of the new score to that of the Alvarado score.

## Materials and Methods

### Patients

This study was approved by the Institutional Review Boards of each participating hospital (Asan Medical Center and Ulsan University Hospital), and written informed consent was obtained from the enrolled patients or guardians on behalf of the participants. We conducted a prospective observational study of consecutive patients who visited the ED of two large, urban, tertiary referral hospitals with symptoms suggestive of acute appendicitis from January 1^st^ to August 31^st^, 2015. All patients who presented to the ED with abdominal pain and right lower quadrant direct tenderness, and who underwent contrast enhanced abdominal CT were enrolled. Patients younger than 17 years, those who were pregnant, and those with renal insufficiency and other contraindications for contrast-enhanced CT scans were excluded from the analysis.

Standard data including demographic, clinical, and laboratory information were collected. The Alvarado score was retrospectively calculated after the end of data collection, and was not used to help predict the likelihood of acute appendicitis. After completing the data entry, abdominal CT with intravenous non-ionic contrast material was performed in all patients and reviewed by board-certified attending radiologists. The decision to operate was made by the surgeon on duty on the basis of clinical impression and abdominal CT scan results. The main outcome was the presence or absence of acute appendicitis based on surgical findings. Appendicitis was considered complicated when perforation or a periappendiceal abscess was present. Diagnoses of patients who did not undergo surgical exploration were made by CT findings. Final diagnoses were classified into three groups: normal appendix, uncomplicated appendicitis, and complicated appendicitis.

Physical examinations including rebound tenderness, defense, psoas, Rovsing’s, obturator sign, and the heel drop test were performed by emergency physicians on duty, after completing education and training for standardized physical examinations for the study. Patients were asked to look at the face of the physician running the test and come down with all his/her weight on his/her heels after standing on his/her toes on a smooth surface [6, 7]. During this exercise, findings indicative of perceived pain were evaluated as a positive result in the heel drop test.

### A new clinical score derivation

The diagnostic score was constructed by backward logistic regression analysis including variables in the Alvarado score and the heel drop test, along with other clinical data with statistical significance. Points for the score were weighted by the odds ratio (OR) rounded to the nearest integer. A new score was calculated for each patient by summing the weighted scores when variables were present.

### Statistical analysis

Frequency tables for categorical variables were calculated, along with the mean ± standard deviation or median (interquartile range) for continuous variables. The results from the logistic regression analysis are presented as ORs and corresponding 95% confidence intervals (CIs). The calibration of the model for goodness-of-fit was performed using the Hosmer-Lemeshow test. The scores were compared by receiver operating characteristic (ROC) analysis, and the area under the ROC curve (AUC) was determined. Two different cutoff values were determined using ROC analysis: one focusing on high sensitivity and another focusing on high specificity. Sensitivity, specificity, negative predictive value (NPV), positive predictive value (PPV), and positive and negative likelihood ratios (PLR, NLR) were calculated for each parameter, calculated new scores, and Alvarado scores. All statistical analyses were performed using SPSS version 21.0 (SPSS, Chicago, IL).

## Results

Of the 292 enrolled patients, 124 (42.5%) were men. The mean age was 37.2 years (range: 17 to 87 years). The total number of patients who had acute appendicitis was 165 (56.5%), including 43 (14.7%) complicated cases. Other diagnoses included 32 (11.0%) non-specific abdominal pain, 22 (7.5%) diverticulitis, 13 (4.5%) pelvic inflammatory disease, 11 (3.8%) ureteral stone, 10 (3.4%) small bowel ileus, 10 (3.4%) colitis, 10 (3.4%) enteritis, and 10 (3.4%) ovarian cyst with or without torsion. The remaining 9 patients had other specific diagnoses.

Patients with acute appendicitis were older than those with other diagnoses. The ratio of having acute appendicitis was higher in men than in women, and in cases with pain migration compared to cases without pain migration. White blood cell (WBC) count, proportion of neutrophils, and C-reactive protein concentrations were higher and rebound tenderness, positive psoas sign, Rovsing sign, and heel drop test were significantly more common in patients with acute appendicitis than in those with other diagnoses. Meanwhile, no significant difference was found in the risk of appendicitis between cases with and without anorexia, nausea or vomiting, muscular defense, or obturator sign. Body temperature was also not significant between the groups (Table 1). Features in the Alvarado score and the heel drop test were analyzed between patients with complicated and uncomplicated appendicitis; however, none of these features showed a significant difference (Table 2).

**Table 1:** Demographic and clinical data for the study population.

Features (n, %)	All (n = 292)	Acute appendicitis (n = 165)	Non-appendicitis (n = 127)	P
Age* (years)	37.2 ± 15.6	39.1 ± 16.4	34.7 ± 14.1	0.015
Male gender	124 (42.5)	81 (49.1)	43 (33.9)	0.009
BT (°C)	36.9 ± 0.7	36.9 ± 0.7	36.8 ± 0.6	0.199
Symptoms				
Anorexia	101 (34.6)	60 (36.4)	41 (32.3)	0.467
Nausea or vomiting	138 (47.3)	86 (52.1)	52 (40.9)	0.058
Migration	109 (37.3)	80 (48.5)	29 (22.8)	<0.001
Laboratory finding**				
WBC (x10^3^/μL)	11.2 (8.1–14.7)	13.1 (10.4–16.1)	8.6 (6.7–11.9)	<0.001
Neutrophil (%)	77.1 (67.4–83.6)	80.0 (73.0–84.8)	71.2 (59.9–81.4)	<0.001
C-reactive protein (mg/dL)	0.6 (0.1–3.2)	1.0 (0.2–3.9)	0.4 (0.1–2.6)	0.002
Physical examination				
Rebound tenderness	144 (49.3)	97 (58.8)	47 (37.0)	<0.001
Defense	71 (24.3)	47 (28.5)	24 (18.9)	0.058
Psoas	88 (30.1)	59 (35.8)	29 (22.8)	0.017
Rovsing	61 (20.9)	42 (25.5)	19 (15.0)	0.029
Obturator	70 (24.0)	46 (27.9)	24 (18.9)	0.075
Heel drop	159 (54.5)	114 (69.1)	45 (35.4)	<0.001

*mean (standard deviation),

**median (interquartile range).

*BT* body temperature; *WBC* white blood cell.

**Table 2:** Features of the Alvarado score and heel drop test in assessing acute appendicitis.

Features (n, %)	Uncomplicated appendicitis (n = 122)	Complicated appendicitis (n = 43)	P
Anorexia	48 (39.3)	12 (27.9)	0.180
Nausea or vomiting	69 (56.6)	17 (39.5)	0.055
Migration	56 (45.9)	24 (55.8)	0.263
Rebound tenderness	69 (56.6)	28 (65.1)	0.327
BT≥37.3°C	24 (19.7)	14 (32.6)	0.084
WBC>10,000/μL	93 (76.2)	33 (76.7)	0.946
Shift to left	84 (68.9)	31 (72.1)	0.691
Heel drop	81 (66.4)	33 (76.7)	0.207

*BT* body temperature; *WBC* white blood cell.

**Fig 1:**
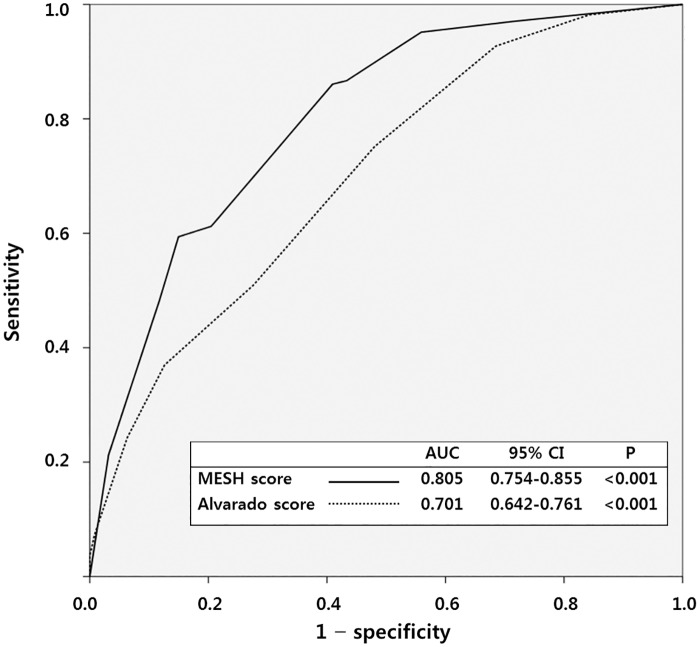
Receiver operating characteristic (ROC) curve analysis for the MESH score and the Alvarado score. *MESH* Migration, Elevated WBC, Shift to left, and Heel drop test; *AUC* area under ROC curve; *CI* confidence interval.

**Fig 2:**
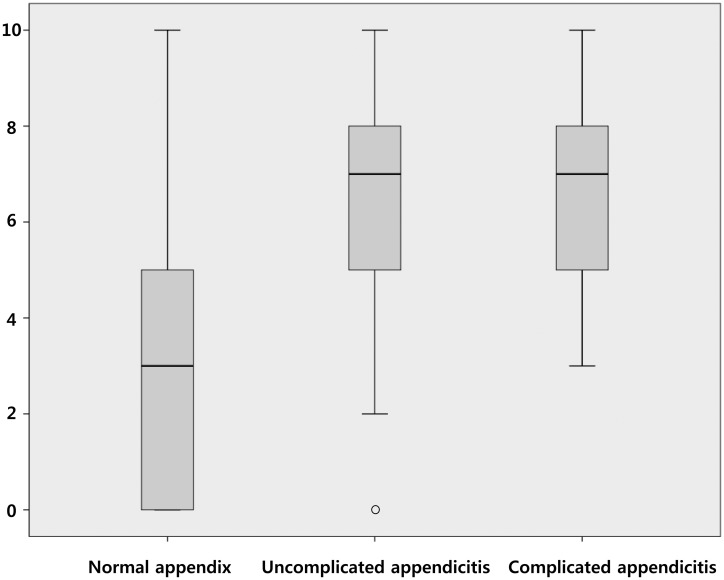
Box plot displaying the distribution of the MESH score in patients with a normal appendix, uncomplicated appendicitis, and perforated appendicitis (cutoff of 5). *MESH* Migration, Elevated WBC, Shift to left, and Heel drop test.

Variables for the new score and the respective points are presented in Table 3. Pain migration [OR 2.44 (95% CI 1.36–4.38), 2 points], positive heel drop test [OR 3.43 (95% CI 1.98–5.93), 3 points], WBC>10,000/μL [OR 3.38 (95% CI 1.87–6.14), 3 points], and shift to left [OR 2.35 (95% CI 1.30–4.25), 2 points] were variables included in the new score, named the MESH (***M***igration, ***E***levated WBC, ***S***hift to left, and ***H***eel drop test) score. The Hosmer—Lemeshow test showed that the model fitted the data well (P = 0.949). The total points of the score ranged from 0 to 10. The new score was compared to the Alvarado score using AUC analysis. In this comparison, the MESH score had a higher AUC than the Alvarado score: [AUC 0.805 (95% CI 0.754–0.855) vs. AUC 0.701 (95% CI 0.642–0.761)] (Fig 1). Based on ROC analysis, scores of 5 and 11 were chosen as cut-off values. A box plot showed that a score of 5 accurately differentiated patients with and without acute appendicitis, regardless of the complications present (Fig 2).

**Table 3:** Development of the MESH score.

Variable	OR (95% CI)	Point
Migration	2.44 (1.36–4.38)	2
Heel Drop	3.43 (1.98–5.93)	3
WBC>10,000/μL	3.38 (1.87–6.14)	3
Shift to left	2.35 (1.30–4.25)	2
Total		10

*MESH* Migration, Elevated WBC, Shift to left, and Heel drop test; *OR* odds ratio; *CI* confidence interval; *WBC* white blood cell.

To determine the predictive characteristics of each parameter, the MESH score and Alvarado score, sensitivity, specificity, PPV, NPV, PLR, and NLR were calculated. The heel drop test had higher sensitivity and specificity than rebound tenderness. A MESH score ≥5 showed higher sensitivity, specificity, PPV, NPV, and PLR, and lower NLR compared to an Alvarado score ≥5, while similar results were shown when the MESH score ≥8 was compared to the Alvarado score ≥7 (Table 4). For subgroup analysis, we compared the diagnostic values of the two scores between genders. Using the AUC, we found that both scores overpredicted for female patients [MESH score: AUC 0.804 (95% CI 0.738–0.870), Alvarado score: AUC 0.731 (95% CI 0.656–0.806)] compared to male patients [MESH score: AUC 0.788 (95%CI 0.702–0.873), Alvarado score: AUC 0.657 (95% CI 0.555–0.758)]. However, the MESH score showed a higher AUC than the Alvarado score, regardless of gender.

**Table 4:** Performance of the parameters included in the Alvarado score, heel drop test, calculated MESH score, and the Alvarado score*.

	Sensitivity	Specificity	PPV	NPV	PLR	NLR
***Parameters***						
Migration	0.48 (0.41–0.56)	0.77 (0.68–0.84)	0.73 (0.64–0.81)	0.53 (0.46–0.60)	2.11 (1.48–3.00)	0.67 (0.57–0.78)
Anorexia	0.36 (0.29–0.44)	0.67 (0.59–0.76)	0.59 (0.49–0.69)	0.45 (0.38–0.52)	1.13 (0.82–1.56)	0.94 (0.83–1.06)
Nausea/vomiting	0.52 (0.44–0.60)	0.59 (0.50–0.68)	0.62 (0.54–0.70)	0.49 (0.41–0.57)	1.27 (0.99–1.64)	0.81 (0.68–0.96)
WBC>10,000	0.76 (0.69–0.82)	0.64 (0.55–0.72)	0.73 (0.66–0.80)	0.68 (0.52–0.76)	2.11 (1.65–2.70)	0.39 (0.28–0.49)
Shift to left	0.70 (0.62–0.76)	0.62 (0.53–0.71)	0.71 (0.63–0.77)	0.61 (0.52–0.70)	1.84 (1.44–2.36)	0.49 (0.38–0.62)
BT≥37.3°C	0.23 (0.17–0.30)	0.80 (0.71–0.86)	0.59 (0.46–0.71)	0.44 (0.38–0.51)	1.12 (0.72–1.75)	0.97 (0.89–1.05)
Rebound tenderness	0.59 (0.51–0.66)	0.63 (0.54–0.71)	0.67 (0.59–0.75)	0.54 (0.46–0.62)	1.59 (1.22–2.06)	0.65 (0.54–0.79)
Heel drop	0.69 (0.61–0.76)	0.65 (0.56–0.73)	0.72 (0.64–0.78)	0.62 (0.53–0.70)	1.95 (1.51–2.52)	0.48 (0.38–0.61)
***Scores***						
MESH score ≥5	0.86 (0.80–0.91)	0.60 (0.50–0.68)	0.73 (0.66–0.79)	0.77 (0.67–0.84)	2.10 (1.69–2.61)	0.24 (0.16–0.35)
MESH score ≥8	0.52 (0.44–0.59)	0.91 (0.84–0.95)	0.88 (0.79–0.93)	0.59 (0.52–0.66)	5.45 (3.12–9.53)	0.54 (0.46–0.63)
Alvarado ≥5	0.75 (0.67–0.81)	0.50 (0.41–0.59)	0.65 (0.58–0.72)	0.62 (0.52–0.71)	1.50 (1.24–1.82)	0.50 (0.38–0.66)
Alvarado ≥7	0.37 (0.30–0.45	0.87 (0.80–0.92)	0.79 (0.68–0.87)	0.52 (0.45–0.58)	2.93 (1.78–4.84)	0.72 (0.64–0.81)

*data are presented with a 95% confidence interval.

*MESH* Migration, Elevated WBC, Shift to left, and Heel drop test; *PPV* positive predictive value; *NPV* negative predictive value; *PLR* positive likelihood ratio; *NLR* negative likelihood ratio.

In male patients with a lower cutoff (MESH score ≥5, and Alvarado score ≥5), the MESH score showed higher sensitivity than the Alvarado score (0.90 vs. 0.71). However, in female patients with a lower cutoff, the difference in sensitivities was small (0.82 vs. 0.78). In both male and female patients with higher cutoffs (MESH score ≥8, and Alvarado score ≥ 7), the differences in specificities were small (male: 0.88 vs. 0.83, and female: 0.92 vs. 0.89) (Table 5).

**Table 5:** Performance of the MESH score and the Alvarado score according to gender*.

		Sensitivity	Specificity	PPV	NPV	PLR	NLR
MESH score ≥5	Male	0.90 (0.81–0.95)	0.49 (0.34–0.64)	0.77 (0.67–0.85)	0.72 (0.53–0.87)	1.76 (1.30–2.38)	0.27 (0.10–0.41)
	Female	0.82 (0.72–0.89)	0.64 (0.53–0.74)	0.70 (0.60–0.78)	0.78 (0.66–0.87)	2.30 (1.70–3.12)	0.28 (0.17–0.45)
MESH score ≥8	Male	0.52 (0.41–0.63)	0.88 (0.74–0.96)	0.89 (0.76–0.96)	0.49 (0.38–0.61)	4.46 (1.91–10.44)	0.54 (0.43–0.69)
	Female	0.45 (0.34–0.56)	0.92 (0.83–0.96)	0.84 (0.70–0.93)	0.63 (0.53–0.71)	5.43 (2.57–11.46)	0.59 (0.49–0.73)
MESH ≥5	Male	0.71 (0.60–0.80)	0.48 (0.33–0.64)	0.72 (0.61–0.81)	0.47 (0.32–0.63)	1.39 (1.01–1.93)	0.58 (0.39–0.86)
	Female	0.78 (0.68–0.86)	0.53 (0.42–0.64)	0.63 (0.53–0.72)	0.71 (0.58–0.81)	1.69 (1.31–2.18)	0.40 (0.26–0.61)
MESH ≥7	Male	0.32 (0.22–0.43)	0.83 (0.68–0.92)	0.78 (0.60–0.90)	0.39 (0.29–0.50)	1.97 (0.93–4.16)	0.81 (0.69–0.95)
	Female	0.42 (0.31–0.53)	0.89 (0.80–0.95)	0.79 (0.64–0.89)	0.60 (0.51–0.69)	3.88 (1.99–7.58)	0.65 (0.54–0.78)

*data are presented with 95% confidence interval.

*MESH* Migration, Elevated WBC, Shift to left, and Heel drop test; *PPV* positive predictive value; *NPV* negative predictive value; *PLR* positive likelihood ratio; *NLR* negative likelihood ratio.

## Discussion

The MESH score was found in our current analysis to be more accurate in classifying actual appendicitis patients than the Alvarado score. It also performed better in subgroup analysis for male and female patients. The superior performance of the MESH scores may be attributable to the inclusion of the heel drop test, which is a more objective and less subject to misinterpretation test than the old rebound test [6]. In addition, excluding variables with lower sensitivity such as anorexia and elevated temperature (≥37.3°C) may have contributed to its accuracy. The strength of the MESH score may also be partially due to the use of prospectively collected data of all patients with abdominal pain and right lower quadrant direct tenderness, rather than only those with confirmed acute appendicitis on surgery. In cases that present with abdominal pain without tenderness, referred pain or other causes of abdominal pain should be considered first. We therefore tried to confine patient enrollment to those with more actual cases with suspected acute appendicitis as the first impression i.e., those with right lower quadrant direct tenderness.

Numerous studies have examined the value of the Alvarado score and the modified Alvarado score in the prediction of acute appendicitis [16–18]. A systematic review of published data showed that the score is most useful in ruling out appendicitis, and a score below 5 has a sensitivity of 94–99% for appendicitis not being present [15]. However, a recent study performed at two academic urban EDs in the United States have criticized the low sensitivity of 72% for the low risk Alvarado score as insufficient to safely discharge patients without additional diagnostic testing [19]. In our current study, the results were similar, with a sensitivity of 75% and a specificity of 50% shown in the low risk group according to the Alvarado score.

Several attempts have been made to refine the variables in the Alvarado score. One study from Hungary tried to modify the score for easier utilization by adding ultrasound investigation as a score variable [20]. The authors reported an AUC increase from 0.749 to 0.899 after addition of the ultrasound variable. However, routinely adding imaging results is not always feasible or practical. Another study from Turkey tried to improve the accuracy of the modified Alvarado score by adding ‘tenesmus’ as a variable [21]. However, they classified patients into two groups (score ≥7 vs. <7), and the goal for score utilization was different from current trends (three risk groups), which limited its application.

In our study, the heel drop test was included as a variable and improved the score’s performance compared to the Alvarado score. Few publications have reported an association between the heel drop test and acute appendicitis [22, 23]. To the best of our knowledge, only one study from Turkey has shown improvement in diagnostic accuracy when classical examination methods are accompanied by a positive heel drop test [7]. In that study, a positive heel drop test had an OR of 2.51 for appendicitis. The combination of the presence of right lower quadrant pain, WBC ≥ 11.950/μL, and heal drop test positivity led to an increase in the diagnosis of appendicitis by almost 8.14- and 22.12-fold in men and women, respectively. In this study, we determined that pain migration, WBC>10,000/μL, shift to left, and positive heel drop test increased the acute appendicitis risk by 2.44-, 3.38-, 2.35-, and 3.43-fold, respectively. A positive heel drop test showed the highest OR among the parameters included in the model. However, compared to WBC and shift to left, the heel drop test showed less sensitivity, meaning it is not useful as a single rule-out parameter for acute appendicitis. While the heel drop test was originally introduced to evoke peritoneal irritation by moving intraperitoneal contents up and down and to detect the presence or absence of peritonitis, especially in acute appendicitis, it is also considered the most sensitive test for meningitis [24]. Although it is similar to rebound tenderness, the heel drop test may be easier to elicit tenderness when the patient has firm abdominal wall muscles. A modified heel drop test can be performed by hitting the bottom of the patient’s heel with the examiner’s hand while the patient remains in the supine position [25]. This procedure will transmit a vibration to the patient’s inflamed peritoneum and elicit right lower quadrant pain.

Our study has several limitations. Although this was a dual-center study with different patient populations, it is possible that our results may not be generalizable to other settings. Since we only enrolled patients on whom the first impression was acute appendicitis (positive right lower quadrant direct tenderness), this score may not be applicable to appendicitis patients without right lower quadrant tenderness, whose diagnoses are usually made by using advanced imaging modalities including ultrasound or computed tomography. The duration of abdominal pain before ED presentation was not reported for our patients, and this could certainly have impacted the patient’s clinical presentation as well as their laboratory results, such as WBC count and its differential [26]. In addition, the interrater reliability for different variables was not verified in our present study, and variation in the interpretation of physical examination findings could have existed. However, we attempted to mitigate this effect via education and training to perform standardized physical examinations before conducting this study. Finally, the true diagnosis of appendicitis in the group of non-operated patients could have been undetected and therefore the reliability of the MESH score on these patients cannot be evaluated. However, on discharge from the ED, patients are always encouraged to revisit when their symptoms get worse, and during the study period, no patients had two or more visits to our ED.

## Conclusions

In summary, we aimed to determine the impact of the heel drop test on the diagnosis of acute appendicitis. The heel drop test showed higher predictive characteristics compared to the rebound tenderness. By combining various significant parameters, the MESH (migration, elevated WBC, shift to left, and heel drop test, leukocytosis, and shift to left) score was found to predict acute appendicitis more accurately than the Alvarado score. Our observations warrant future studies to refine the variables and its cutoffs, and validations to improve the clinical diagnosis of acute appendicitis.

## Supporting Information

S1 Table (XLSX)
